# Exertional heat stroke with multiorgan dysfunction in a community sports event: case report and one-month follow-up

**DOI:** 10.1186/s12245-025-01071-3

**Published:** 2025-11-21

**Authors:** Ninh Xuan Nguyen, Ngoc Tien Pham, Huong Thi Thanh Le, Quoc Viet Tran, Hang Ngoc Thuy Tran, Thi Kim Thanh Vo, Phong Van Phan

**Affiliations:** 1 Emergency Department, Vinmec Central Park International Hospital, Vinmec Healthcare System, Ho Chi Minh City, Vietnam; 2 Intensive Care Unit, Vinmec Central Park International Hospital, Vinmec Healthcare System, Ho Chi Minh City, Vietnam

**Keywords:** Exertional heat stroke, Multiorgan dysfunction, Rhabdomyolysis, Cardiac injury, Low- and middle-income countries, Emergency preparedness

## Abstract

**Background:**

Exertional heat stroke (EHS) is a life-threatening emergency characterized by central nervous system (CNS) dysfunction with hyperthermia and frequent multiorgan injury. Although outcomes have improved with rapid on-site cooling in elite sports and military contexts, data from community sporting events in low- and middle-income countries (LMICs) remain scarce.

**Case presentation:**

We report a 30-year-old previously healthy Vietnamese male who collapsed during a 5-km community run. He presented with encephalopathy, generalized seizure, metabolic acidosis, acute kidney injury (AKI) (creatinine 149 µmol/L, estimated glomerular filtration rate (eGFR) 53), rhabdomyolysis (creatine kinase (CK) 3745 U/L), and myocardial injury (high-sensitivity troponin I (hs-troponin I) peak 3.87 ng/mL). The electrocardiogram (ECG) showed incomplete right bundle branch block with minor ectopy, while echocardiography revealed preserved systolic function. With rapid cooling and supportive care, renal, muscular, and cardiac abnormalities resolved. At one-month follow-up, hs-troponin I and CK had normalized (0.0016 ng/mL; 155 U/L), creatinine was 81 µmol/L, and ECG was normal, confirming full clinical recovery.

**Conclusion:**

This case illustrates EHS with multiorgan dysfunction successfully managed at a community sporting event in Vietnam. The comprehensive one-month biomarker and cardiac follow-up highlights the potential for full recovery, while emphasizing the need for cost-effective emergency preparedness-including trained staff and rapid cooling strategies-in LMIC community events.

## Introduction

Exertional heat stroke (EHS) is a life-threatening emergency defined by central nervous system (CNS) dysfunction with core temperature often but not always ≥ 40 °C, frequently accompanied by rhabdomyolysis, acute kidney injury, and myocardial injury [1, 2]. Because body temperature may decline before measurement, diagnosis depends on both clinical and systemic features. Mortality remains high if recognition and cooling are delayed [2].

Rapid prehospital cooling is the cornerstone of management. International guidelines recommend cold-water immersion or equivalent methods initiated on site, followed by supportive care, which have improved outcomes in elite sports and military populations [3].

However, most evidence derives from high-resource settings, while data from community events in low- and middle-income countries (LMICs) are scarce [4]. With growing popularity of endurance races in these regions but limited preparedness, delayed response may increase risk.

We report a 30-year-old man in Vietnam who developed EHS during a 5-km run. This case is notable for occurring in a community LMIC setting and for its comprehensive follow-up, including serial high-sensitivity troponin, creatine kinase, and renal markers, confirming complete recovery at one month. It also highlights the need for feasible early recognition and on-site cooling strategies in community sports, making this case a valuable contribution to clinical care and public health preparedness.

## Case presentation

A 30-year-old previously healthy Vietnamese male collapsed during the final stretch of a 5-km community run in July 2025. On-site staff initiated cooling with ice packs and cold towels and infused 1 L of Ringer’s lactate before transfer.

### Emergency department (ED; day 0)

The patient was confused, agitated, and developed a generalized tonic–clonic seizure treated with intravenous midazolam. Vital signs: BP 109/72 mmHg, HR 130 bpm, RR 24/min, T 38.5 °C (measured tympanically; rectal thermometry not performed after partial cooling), SpO₂ 96% (room air). The measured value may underestimate true core temperature, given that active cooling had already been initiated before hospital transfer. He was diaphoretic and hot to touch. Initial labs showed metabolic acidosis (pH 7.24, HCO₃⁻ 14.8 mmol/L, lactate 8.06 mmol/L), acute kidney injury (AKI) (creatinine 149 µmol/L, eGFR 53), elevated hs-troponin I (0.38 ng/mL), CK 247 U/L, CK-MB 34.4 U/L, and mild hypokalemia (3.37 mmol/L). He was diagnosed with EHS complicated by encephalopathy, rhabdomyolysis, AKI, and possible myocardial injury. Immediate cooling and IV fluids were administered.

Key laboratory and cardiac findings across the clinical course are summarized in Table 1.

**Table 1:** Key laboratory and cardiac findings across clinical course

Timepoint	Lactate (mmol/L)	Creatinine (µmol/L)	CK (U/L)	hs-Troponin I (ng/mL)	Cardiac findings
ED (Day 0)	8.06	149 (eGFR 53)	247	0.38	Seizure; sinus tachycardia
ICU peak (12 h)	–	149 → 86	3745	3.87	Incomplete RBBB; PACs; EF 60%
Day 2	< 1.0	86	–	1.99	Clinically stable
Cardiology (D3–4)	–	80	3245	0.29	Infrequent ectopy; sinus rhythm
1-month follow-up	–	81 (eGFR 112)	155	0.0016	Normal ECG; asymptomatic

Abbreviations: CK, creatine kinase; eGFR, estimated glomerular filtration rate; hs-Troponin I, high-sensitivity troponin I; ED, Emergency Department; ICU, Intensive Care Unit; EF, ejection fraction; ECG, electrocardiogram; RBBB, right bundle branch block; PACs, premature atrial contractions

Serial laboratory values and cardiac assessments showing multiorgan involvement at presentation with progressive normalization and complete recovery by one month

### Intensive care unit (ICU; days 0–2)

hs-troponin I peaked at 3.87 ng/mL (12 h) and CK rose to 3745 U/L, while creatinine gradually normalized. ECG showed sinus rhythm with incomplete right bundle branch block and occasional premature atrial contractions (PACs); echocardiography revealed preserved LV systolic function (ejection fraction (EF) 60%). By day 2, lactate had normalized and the patient was fully conscious.

### Cardiology ward (Days 3–4)

Biomarkers declined (hs-troponin I 0.29 ng/mL, CK 3245 U/L, creatinine 80 µmol/L). Holter monitoring showed only infrequent supraventricular and ventricular ectopy. He remained hemodynamically stable and asymptomatic, and was discharged on day 4.

### Follow-up (1 month)

At clinic review (August 9, 2025), the patient was asymptomatic with full recovery. Labs showed CK 155 U/L, CK-MB 40.9 U/L, hs-troponin I 0.0016 ng/mL, creatinine 81 µmol/L, eGFR 112; ECG was normal. He was cleared for gradual return to exercise.

**Fig. 1:**
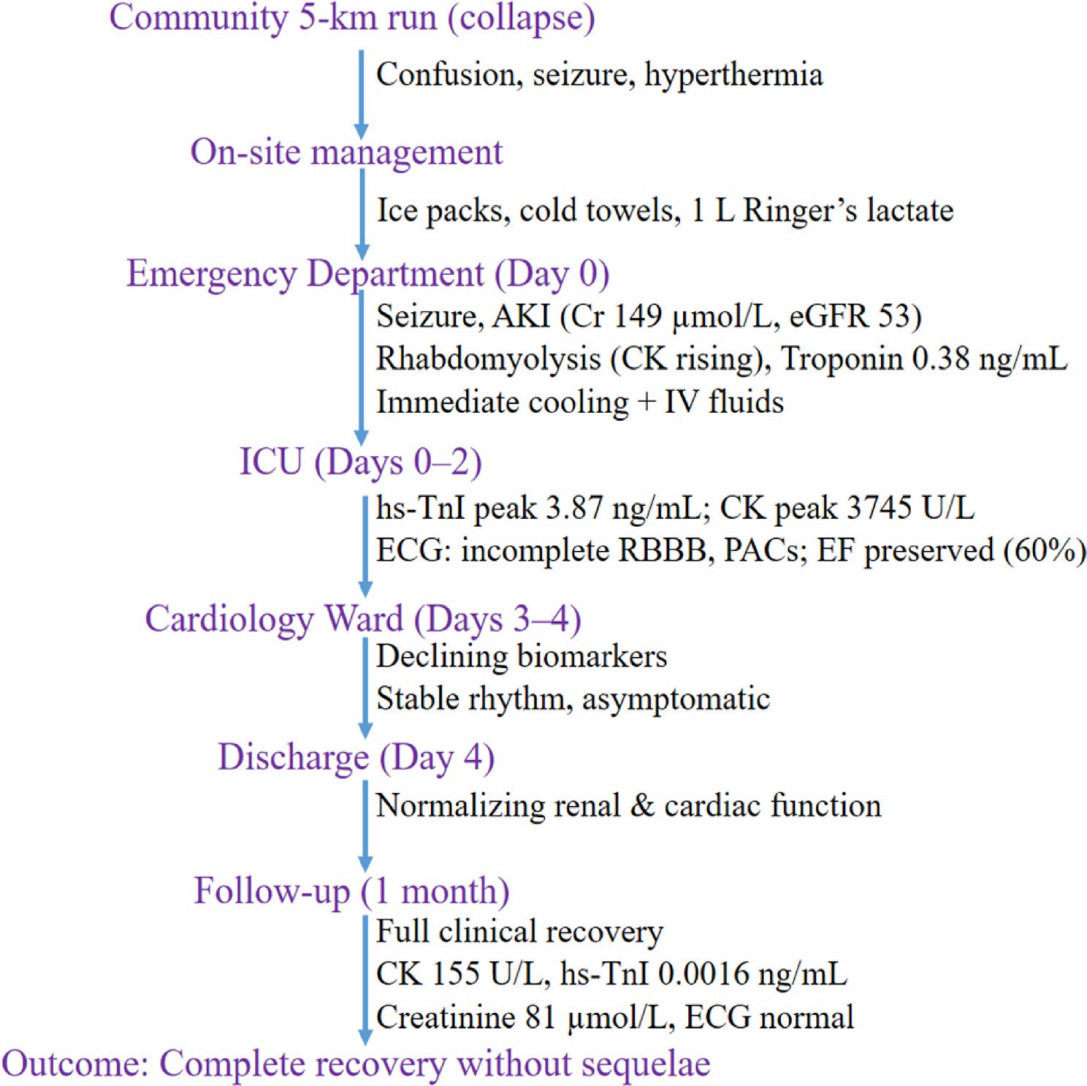
Flow diagram of clinical course and outcomes. Clinical pathway from exertional heat stroke onset to complete recovery at one month

The overall clinical pathway from collapse to full recovery is illustrated in Fig. 1.

## Discussion

### General overview of EHS

EHS is a time-critical emergency defined by central nervous system dysfunction (confusion, seizure, coma) with core temperature often but not always >40 °C, since cooling or delays may lower measured values [2]. In our case, the admission temperature was 38.5 °C, likely underestimated because active cooling had already been initiated; rectal thermometry is considered the gold standard, and values can decline rapidly after collapse [2, 5]. Diagnosis in this case was supported by exertional collapse, CNS dysfunction, and multiorgan injury, consistent with accepted criteria for EHS and World Athletics guidelines [2, 5, 6]. Unlike classic heat stroke, which affects vulnerable individuals during heat waves, EHS occurs in otherwise healthy people during intense exertion, most often in hot and humid environments but it can also develop in moderate climates depending on exertional intensity and individual susceptibility [1, 2].

Pathophysiology involves failure of thermoregulation with direct thermal cytotoxicity, systemic inflammation, endothelial injury, and coagulopathy, leading to multiorgan dysfunction such as rhabdomyolysis, acute kidney injury, hepatic injury, and cardiovascular complications [2, 5].

Rapid recognition and immediate cooling are vital. Mortality may reach 30% in delayed or complicated EHS and 80% in classic cases, but declines markedly when cold-water immersion is initiated promptly after collapse, forming the cornerstone of current prehospital and in-hospital management [2, 7]. Cold-water immersion (CWI) remains the gold standard method for rapid cooling, as endorsed by World Athletics and supported by meta-analyses showing superior survival compared with other techniques [3, 8]. Simple on-site CWI setups, including ice baths or tarp-assisted immersion with locally available water, should be mandatory for all events where heatstroke risk exists, as they are low-cost, accessible, and lifesaving even in resource-limited settings.

### Cardiac involvement in EHS

Cardiac injury is increasingly recognized in EHS. Mechanisms include direct thermal damage, systemic inflammation, catecholamine surge, and endothelial dysfunction, which may trigger ischemia, arrhythmias, or transient systolic impairment [2, 5]. Clinically, this often appears as troponin elevation, conduction changes, or tachy/bradyarrhythmias during the acute phase [2, 5].

Troponin rise is common and usually reflects reversible stress-related injury, though in some cases it may unmask acute coronary syndrome [2, 5, 9]. Whiticar et al. described a young man with EHS and transient troponin I elevation that normalized [10]. Population data from the 2003 European heatwave also linked troponin increases with worse prognosis, with 28-day mortality reaching 58% in the Lyon cohort [11].

Our patient showed the typical transient phenotype: hs-troponin I peaked at 3.87 ng/mL and CK at 3745 U/L, yet echocardiography confirmed preserved EF (60%) and Holter revealed only mild ectopy with incomplete RBBB. By one month, all markers normalized (hs-TnI 0.0016 ng/mL; CK 155 U/L), and he was asymptomatic, aligning with prior reports [9, 10]. Alternative diagnoses such as acute coronary syndrome or myocarditis were considered but were unlikely in this young, previously healthy patient without chest pain, regional wall motion abnormalities, or persistent ECG changes. The rapid decline in hs-troponin I also favored transient stress-related injury. Advanced imaging (cardiac MRI or coronary CT angiography) would be appropriate in cases with markedly higher troponin levels, persistent arrhythmias, or ongoing symptoms, but was not deemed essential here. In LMIC contexts where such resources may be limited, careful serial biomarker measurement, ECG/Holter monitoring, and echocardiography represent pragmatic and cost-effective alternatives.

### Multiorgan dysfunction and recovery trajectory

Severe EHS frequently leads to, but does not require, multiorgan dysfunction, which may include rhabdomyolysis, AKI, hepatic involvement, and CNS impairment. The underlying pathophysiology involves direct heat cytotoxicity, systemic inflammation, and endothelial and coagulation disturbances, resulting in potential muscle necrosis, myoglobinuria, renal, hepatic, or cardiovascular failure [1, 2, 5, 12]. In intensive care settings, management focuses on rapid temperature control and organ support, as detailed by Stomeo et al. [12].

Mortality varies by context: in severe ICU or heatwave cohorts, rates can exceed 20–50%, especially in classic heat stroke, while EHS generally carries lower but significant risk when complicated by AKI, DIC, or shock [5, 11, 13]. Beyond short-term mortality, survivors may suffer long-term sequelae—functional decline (Argaud 2007) [11], higher risk of chronic kidney disease (Tseng et al., 2020; adjusted HR = 4.35, 95% CI 2.83–6.68 [14]), and persistent neurocognitive deficits [15].

Our patient demonstrated simultaneous dysfunction (rhabdomyolysis CK >3700 U/L, AKI, conduction changes, and encephalopathy) but achieved complete recovery, with normalization of biomarkers and no residual deficits at one month. Such outcomes are less often documented, as most reports focus on acute hospitalization without extended follow-up [11, 14, 15]. Nonetheless, long-term mortality and morbidity after severe heatstroke have been recognized in the literature, including risks of chronic kidney disease, neurocognitive sequelae, and functional decline [11, 14, 15], underscoring the need for ongoing surveillance beyond the initial recovery period. This case thus adds rare longitudinal evidence - particularly in a community sports setting in a low- and middle-income country - that multiorgan dysfunction in EHS can be fully reversible with rapid recognition and supportive care.

### Public health and LMIC context

Most reports on EHS come from elite athletes and military cohorts in high-resource countries, creating a clear geographic bias. A systematic review found that 71% of exertional heat illness studies were from the United States, with minimal representation from LMICs [16]. A scoping review of endurance running confirmed that evidence largely comes from marathons in high-income settings, leaving field-based data from LMICs scarce [4]. Available literature also suggests that emergency preparedness and structured response capacity remain underdeveloped in many LMICs, which may delay recognition and definitive cooling in community events [4, 16].

Our case illustrates this gap by documenting EHS at a community event in Vietnam, where limited sports medicine infrastructure risks delayed recognition and suboptimal emergency response. This case also underscores the diagnostic challenge in LMICs, where rectal thermometry is often unavailable, reinforcing the need to rely on the full clinical picture rather than a single temperature threshold. Practical, cost-effective measures may include tarp-assisted immersion with locally available cold water or large-volume ice packs when cold-water immersion tanks are not feasible, consistent with International Olympic Committee recommendations [3, 4]. Such simple strategies should be integrated into local event planning and emergency preparedness to improve outcomes in resource-limited settings.

### Study limitations

This report has several limitations. First, it describes a single case, which limits generalizability. Second, advanced cardiac imaging such as cardiac MRI or coronary CT angiography was not performed; however, the diagnostic work-up—including serial ECGs, cardiac biomarkers, and echocardiography—was comprehensive and consistent with current EHS management recommendations [2, 5, 8]. Given the patient’s age, clinical profile, absence of chest pain, normal echocardiographic findings, and rapid biomarker normalization, significant structural or ischemic heart disease was unlikely. In high-resource settings, advanced imaging would be indicated only for markedly elevated troponin levels, persistent ECG changes, or ongoing symptoms. In LMIC contexts, where such modalities may not be readily available, pragmatic strategies such as serial biomarkers, ECG/Holter monitoring, and echocardiography remain appropriate for clinical decision-making and follow-up. Third, hepatic, coagulative, and respiratory parameters were not systematically assessed, although no clinically significant abnormalities were observed during hospitalization, which represents an additional limitation. Finally, although this case highlights the value of longitudinal monitoring with confirmation of full recovery, it cannot claim absolute novelty, since a few prior reports have also documented extended follow-up after EHS. Nonetheless, such data remain rare in community sporting events and LMIC settings, where our report adds practical relevance.

### Clinical implications & future directions

This case underscores key lessons in EHS. Early recognition and rapid cooling are lifesaving, while serial biomarker and cardiac monitoring help differentiate transient myocardial stress from structural injury and guide safe return to activity.

At the public health level, it highlights the preparedness gap in LMIC community events, where limited sports medicine resources increase risks. Implementing trained on-site staff, cold-water immersion, and clear transfer protocols should be prioritized to reduce preventable complications.

Future work should focus on prospective data collection in LMICs to better characterize epidemiology, cardiac risks, and long-term outcomes of EHS, thereby strengthening global guidelines and preventive strategies.

## Conclusion

This case demonstrates that EHS can precipitate severe multiorgan dysfunction, including transient cardiac involvement, yet full recovery is achievable with rapid recognition, early cooling, and supportive care. While advanced imaging such as cardiac MRI or coronary CT angiography would provide greater diagnostic certainty, the patient’s age, absence of chest pain, preserved ventricular function, and rapid biomarker normalization supported reversible myocardial stress injury. In LMIC settings, where advanced modalities are often unavailable, structured serial assessment - including biomarkers, ECG/Holter, and echocardiography - offers a practical and reliable alternative to guide safe recovery. Beyond the individual outcome, this case highlights the vulnerability of community sporting events in resource-limited contexts, underscoring the urgent need for preparedness through trained staff and simple, locally available cooling methods to reduce preventable morbidity and mortality.

### Learning points

- EHS can cause life-threatening multiorgan dysfunction, including rhabdomyolysis, acute kidney injury, and transient cardiac involvement, even in previously healthy young adults.
- Early recognition and rapid cooling, ideally cold-water immersion initiated immediately at the onset of symptoms, are critical to survival and functional recovery.
- Cardiac biomarker elevation in EHS often reflects reversible stress injury, but structured follow-up with ECG, Holter, and echocardiography is necessary to exclude persistent damage.
- Community sporting events in LMICs remain underprepared, underscoring the need for trained staff, on-site cooling capacity, and clear referral pathways.
- Longitudinal follow-up with standardized biomarkers provides valuable evidence of recovery and should be incorporated into clinical practice and future studies.

## Data Availability

All relevant clinical data supporting the findings of this case are included within the article.
